# Genetic Diversity, Haplotype Relationships, and *kdr* Mutation of Malaria *Anopheles* Vectors in the Most *Plasmodium knowlesi*-Endemic Area of Thailand

**DOI:** 10.3390/tropicalmed7120412

**Published:** 2022-12-01

**Authors:** Tanawat Chaiphongpachara, Sedthapong Laojun, Tanasak Changbunjong, Suchada Sumruayphol, Nantana Suwandittakul, Sakultip Chookaew, Yuppayong Atta

**Affiliations:** 1 Department of Public Health and Health Promotion, College of Allied Health Sciences, Suan Sunandha Rajabhat University, Samut Songkhram 75000, Thailand; 2 Department of Pre-Clinic and Applied Animal Science, Faculty of Veterinary Science, Mahidol University, Nakhon Pathom 73170, Thailand; 3 The Monitoring and Surveillance Center for Zoonotic Diseases in Wildlife and Exotic Animals (MoZWE), Faculty of Veterinary Science, Mahidol University, Nakhon Pathom 73170, Thailand; 4 Department of Medical Entomology, Faculty of Tropical Medicine, Mahidol University, Bangkok 10400, Thailand; 5 Vector Borne Disease Control Center 11.5, Ranong 85000, Thailand

**Keywords:** genetic diversity, *VGSC* mutation-mediated knockdown resistance, *Anopheles*, mosquitoes, *Plasmodium knowlesi*

## Abstract

*Plasmodium knowlesi,* a malaria parasite that occurs naturally in long-tailed macaques, pig-tailed macaques, and banded leaf monkeys, is currently regarded as the fifth of the human malaria parasites. We aimed to investigate genetic diversity based on the cytochrome *c* oxidase subunit I (*COI*) gene, detect *Plasmodium* parasites, and screen for the voltage-gated sodium channel (*VGSC*)-mutation-mediated knockdown resistance (*kdr*) of *Anopheles* mosquitoes in Ranong province, which is the most *P. knowlesi*-endemic area in Thailand. One hundred and fourteen *Anopheles* females belonging to eight species, including *An. baimaii* (21.05%), *An. minimus* s.s. (20.17%), *An. epiroticus* (19.30%), *An. jamesii* (19.30%), *An. maculatus* s.s. (13.16%), *An. barbirostris* A3 (5.26%), *An. sawadwongporni* (0.88%), and *An. aconitus* (0.88%), were caught in three geographical regions of Ranong province. None of the *Anopheles* mosquitoes sampled in this study were infected with *Plasmodium* parasites. Based on the sequence analysis of *COI* sequences, *An. epiroticus* had the highest level of nucleotide diversity (0.012), followed by *An. minimus* (0.011). In contrast, *An. maculatus* (0.002) had the lowest level of nucleotide diversity. The Fu’s *Fs* and Tajima’s *D* values of the *Anopheles* species in Ranong were all negative, except the Tajima’s *D* values of *An. minimus* (0.077). Screening of *VGSC* sequences showed no presence of the *kdr* mutation of *Anopheles* mosquitoes. Our results could be used to further select effective techniques for controlling *Anopheles* populations in Thailand’s most *P. knowlesi*-endemic area.

## 1. Introduction

Four species of malaria parasite have long been known to cause human health issues, including *Plasmodium vivax*, *P. ovale*, *P. malariae*, and *P. falciparum* [1]. *Plasmodium knowlesi*, a malaria parasite that occurs naturally in long-tailed macaques (*Macaca fascicularis*), pig-tailed macaques (*Ma. nemestrina*), and banded leaf monkeys (*Presbytis melalophos*), is now regarded as the fifth human malaria parasite [2,3]. The first naturally acquired human infection was documented in 1965 when a traveler acquired *P. knowlesi* after a brief stay in peninsular Malaysia [4]. Human *P. knowlesi* infections are prevalent in Southeast Asian countries such as Thailand, Cambodia, Myanmar, the Philippines, Singapore, Vietnam, Malaysia, and Indonesia. [3]. In addition, this zoonotic malaria parasite has also been reported in other regions after being carried by travelers who visited Southeast Asian countries such as Malaysia [5,6] and Thailand [7,8].

Mosquitoes of the genus *Anopheles* are responsible for spreading malaria to humans. Although wild *Plasmodium*-infected *Anopheles* mosquitoes have been extensively surveyed to validate their role as malaria vectors, few studies have been able to confirm vectors of *P. knowlesi* due to a lack of appropriate molecular tools [9]. In 1961, *Anopheles hackeri* was identified as the natural vector of simian malaria *P. knowlesi* in peninsular Malaysia, based on sporozoites inoculated into a rhesus monkey [10]. However, this *Anopheles* species cannot transmit *P. knowlesi* to humans because it feeds mainly on monkeys and does not attack humans. The confirmation of *P. knowlesi* vectors using molecular techniques was begun in 2006 by Vythilingam et al. [11], who discovered that *An. latens* is a vector of *P. knowlesi* in Sarawak, Malaysia, using a nested polymerase chain reaction (PCR) assay. In 2011, Marchand et al. [12] reported *P. knowlesi* infections in *An. dirus* sensu stricto (s.s.) in Southern Vietnam. Jiram et al. [13] confirmed that *An. cracens* is a vector of *P. knowlesi* in Kuala Lipis in peninsular Malaysia. In 2009, *P. knowlesi* infections were also found in *An. sundaicus* sensu lato (s.l.) in Katchal Island, India [14]. Recently, *An. balabacensis* and *An. donaldi* were identified as vectors of *P. knowlesi* in Lawas, Northern Sarawak, Malaysian Borneo, based on the detection of *Plasmodium* DNA in the salivary glands of wild *Anopheles* mosquitoes using a nested PCR assay [9]. As noted earlier, *Anopheles* mosquitoes, confirmed to be *P. knowlesi* vectors in the past, are only found in Malaysia, with single reports from Vietnam and India. Therefore, other countries should continue to investigate *Anopheles* mosquito vectors to control knowlesi malaria effectively. Since *Anopheles* vectors behave differently in different regions, a malaria vector in one region may not be a malaria vector in another [15].

Thailand is a malaria-epidemic country, especially in border areas, caused by *P. vivax* and *P. falciparum* [16]. Nevertheless, the trend of *P. vivax* and *P. falciparum* malaria cases is one of annual decrease. Thus, Thailand’s Ministry of Public Health set a goal of eliminating malaria by 2024. However, the surge of knowlesi malaria cases may hinder Thailand’s efforts to eliminate malaria. In 2004, the first case of a human *P. knowlesi* infection was reported in Thailand. The patient had a travel history that included a few weeks in the forest in Prachuap Khiri Khan province [17]. After the first case was reported, there continued to be a few humans infected per year (<10 cases) until 31 cases were reported in 2018. The following year, *P. knowlesi* infection rates remained high (19 cases in 2019), and they began to increase in 2020 (22 cases) and 2021 (72 cases). In January–October 2022, 140 *P. knowlesi* infected patients were reported [18]. Although the number of *P. knowlesi* infected patients is currently on the rise, there is no information available on the natural vectors of *P. knowlesi* in Thailand, which makes controlling the disease difficult.

Ranong is one of Thailand’s southern provinces, near the Myanmar border, and is the most *P. knowlesi*-endemic area in Thailand, with 53 infected patients in 2022 (accounting for 96.36% of total cases during January–October 2022). In contrast, other malaria infections are rare (one case of *P. vivax* and another of *P. falciparum* in 2022). A substantial portion of Ranong is forested area, a vital habitat for the primary malaria vectors in Thailand, including *An. dirus, An. minimus*, and *An. maculatus* [15,16]. Meanwhile, a portion of Ranong is coastal area, which is the habitat of *An. epiroticus*, a secondary malaria vector in Thailand [19]. For malaria control to be successful, comprehensive knowledge of *Anopheles* vectors is necessary [15]. However, in-depth information on *Anopheles* mosquitoes in Thailand’s most *P. knowlesi*-endemic area is still lacking.

The genetic diversity of insect vectors in endemic areas is critical, providing useful information about the taxonomic status of species and the spatial limits of natural populations [20]. This knowledge permits researchers to understand and predict the epidemiology, distribution, and transmission dynamics of vector-borne diseases based on the basic biology of the vectors [20]. The cytochrome *c* oxidase subunit I (*COI*) gene is a frequently utilized marker in molecular studies on the genetic diversity of insects, including *Anopheles* mosquitoes, due to its high accuracy [21,22,23].

In addition, genetic monitoring of *Anopheles* mosquitoes also allows for more effective vector control strategies. Malaria vector control via the use of insecticide-treated nets (ITNs), long-lasting insecticide nets (LLINs), and indoor residual spraying (IRS) of insecticides is the primary technique for reducing malaria transmission [24]. However, insecticide-resistant *Anopheles* mosquitoes have been reported in many countries [25]. The voltage-gated sodium channel (*VGSC*) is the main target for both pyrethroid and dichlorodiphenyltrichloroethane (DDT) insecticides [25]. Molecular studies can help to examine the polymorphisms associated with the resistance of several insects, including *Anopheles* mosquitoes, against pyrethroids and DDT, also called knockdown resistance (*kdr*), based on genetic mutations of codon 1014 in the *VGSC* gene [26,27,28].

To optimize entomological information for vector control strategies in Thailand’s most *P. knowlesi*-endemic area, in-depth molecular information on *Anopheles* mosquitoes is required. The present study aimed to investigate genetic diversity based on *COI*, detect *Plasmodium* parasites, and screen for *VGSC*-mutation-mediated knockdown resistance of *Anopheles* mosquitoes in Ranong province, which is Thailand’s most *P. knowlesi*-endemic area.

## 2. Materials and Methods

### 2.1. Ethics Statement

The current investigation was conducted in compliance with the conditions outlined in the guidelines for animal care and usage in research developed by the Suan Sunandha Rajabhat University in Thailand. The Institutional Animal Care and Use Committee of the Suan Sunandha Rajabhat University in Bangkok, Thailand, reviewed and approved all experimental procedures and fieldwork beforehand (Animal Ethics Permission number: IACUC 64-010/2021).

### 2.2. Study Sites and Sample Collection

We conducted our study in Ranong province, Thailand’s most *P. knowlesi*-endemic area [18]. Ranong is the northernmost province on Thailand’s Andaman coast and shares a border with Myanmar. It is located around 580 km from Bangkok, Thailand. Three different locations in Ranong province were selected for *Anopheles* collection, including northern (10°45′48.3″ N, 98°53′31.4″ E), central (9°57′20.1″ N, 98°42′05.3″ E), and southern (9°22′06.8″ N, 98°27′52.1″ E) areas. The northern part of Ranong province includes the northernmost Kraburi and La-un districts and is covered by mountains and forests. The central part includes high forested hills on the right bank of a large reservoir (Hat Som Paen reservoir), the left bank of which is adjacent to the Andaman Sea. In addition, the central area includes large islands such as Koh Chang and Koh Phayam. The southern area includes the southernmost districts of the province, Kapur and Suk Samran, boundaried on the left side by the Andaman coast and on the right side by high mountain and forest areas. Many natural water sources on the left bank of the central and southern sampling areas are brackish water sources. All three areas of Ranong are knowlesi malaria outbreak zones (a total of 8 cases in 2020–2022 for the northern area or Kraburi and La-un districts; 24 cases for the central area or Mueang Ranong district; and 46 cases for the southern area or Kapur and Suk Samran districts), according to a malaria report from Thailand’s Ministry of Public Health [18].

**Figure 1:**
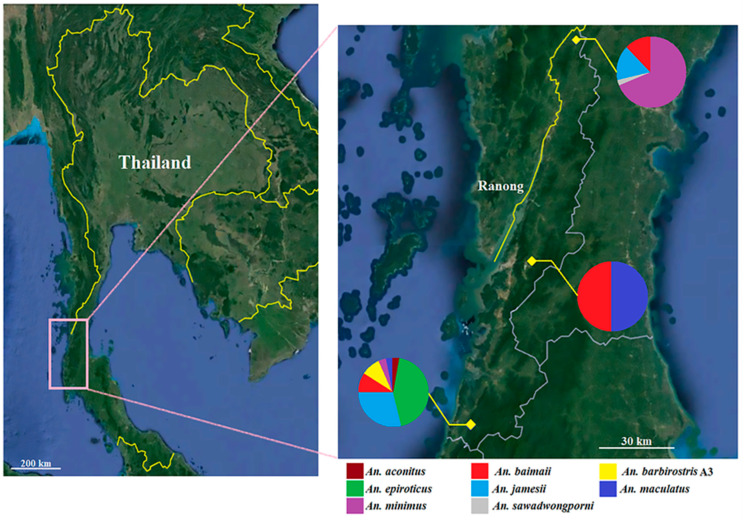
Map of study sites and diversity of *Anopheles* species in Ranong province, southern Thailand. Pie charts in this map show the frequency of each species in each location. This map was obtained from Google Earth Pro v 7.1.8 (https://earth.google.com (accessed on 10 October 2022)).

We conducted adult *Anopheles* collections once every two months between January and June 2022 in accordance with the survey plan of the Ranong Vector Borne Disease Control Center. *Anopheles* mosquitoes from three different locations in Ranong province (Figure 1) were collected throughout the night between 18:00 and 6:00 over five nights, using 12 BG-Pro CDC-style traps (BioGents, Regensburg, Germany) with BG-lure cartridges (BioGents, Regensburg, Germany) and solid carbon dioxide (dry ice). The mosquito bags were removed from the traps in the morning (6:00 a.m.) and kept in the freezer at −20 °C until the mosquitoes died. Then, the gathered mosquito samples were brought to the College of Allied Health Sciences laboratory at Suan Sunandha Rajabhat University in Thailand and stored in the freezer at −20 °C until further use.

### 2.3. Morphological and Molecular Species Identification

The initial identification of wild-caught *Anopheles* mosquitoes at the species/group level was performed via morphological examination under a stereo microscope (Nikon Corp., Tokyo, Japan), using an illustrated key of adult *Anopheles* from Thailand [29]. Each morphologically identified *Anopheles* specimen was kept individually in a 1.5 mL micro-centrifuge tube with silica gel (one specimen/tube) and stored at −20 °C until required. Next, all *Anopheles* specimens were reconfirmed using molecular methods to distinguish sibling species and prevent operator mistakes. Genomic DNA was extracted from the legs of individual *Anopheles* mosquitoes using the FavorPrep™ mini kit (Favorgen Biotech, Ping-Tung, Taiwan) according to the manufacturer’s protocol. Multiplex allele-specific PCR (MAS-PCR) assays based on the internal transcribed spacer 2 (ITS2) region of DNA were used to identify the following: (1) five sibling species of the Dirus complex, including *An. dirus* s.s., *An. baimaii*, *An. cracens*, *An. nemophilous,* and *An. scanloni*; (2) five species of the Maculatus group, including *An. maculatus* s.s., *An. dravidicus, An. pseudowillmori*, *An. rampae*, and *An. sawadwongporni*; and (3) five species of the Funestus group, including *An. minimus* s.s., *An. harrisoni*, *An. aconitus*, *An. pampanai,* and *An. varuna*, according to the previous protocols of Walton et al. [30], Walton et al. [31], and Garros et al. [32], respectively. For the molecular identification of other *Anopheles* species, we compared *COI Anopheles* sequences to the barcode reference library.

### 2.4. Detection of Malaria-Infected Anopheles Mosquitoes

For screening of *Plasmodium* sporozoites in *Anopheles* mosquitoes, the fast COX-I PCR method was used, as described previously by Echeverry et al. [33]. We extracted *Plasmodium* DNA from the head and thorax of each female *Anopheles* mosquitoes. An approximately 520 bp segment of the *Plasmodium* DNA *COI* region was amplified using the primer pair COX-IF (5′ AGA ACG AAC GCT TTT AAC GCC TG 3′) and COX-IR (3′ ACT TAA TGG TGG ATA TAA AGT CCA TCC wGT 5′). The PCR amplifications were conducted using a thermal cycler (Biometra TOne Series, Germany) in a total volume of 25 μL, containing 4 μL of DNA template, a 1 µM concentration of each primer, 1x blood Phusion PCR Master Mix (Thermo Scientific, Waltham, MA, USA), and distilled water up to 25 μL. The PCR reaction conditions were as follows: initial steps at 98 °C at 4 min followed by 70 cycles of 98 °C at 1 s, 69 °C at 5 s, and 72 °C at 35 s, with a final extension at 72 °C at 10 min. Each PCR contained negative (water without DNA) and positive (DNA of *P. falciparum* from culture) controls. PCR products were spread by electrophoresis on 1% agarose gels stained with Midori Green DNA stain (Nippon Gene, Tokyo, Japan), under an ImageQuant LAS 500 imager (GE Healthcare Japan Corp., Tokyo, Japan). A specimen showing a clear DNA band size of 540 bp on agarose gel was considered infectious (*Plasmodium*-genus-positive). If a positive sample had been found, PCR products would have been sent to a service company for DNA sequencing, and then those sequences would have been used for species assessments of *Plasmodium* parasites by comparing them to reference sequences in the Barcode of Life Data System database (BOLD).

### 2.5. Polymerase Chain Reaction (PCR) and Sequencing of COI and VGSC Genes

Genomic DNA derived from the legs of each *Anopheles* specimen was used to amplify *COI* and *VGSC* gene fragments. The PCR amplification of approximately 709 bp of the *COI* gene was performed using two primers, including forward primer COI_F (5′-GGA TTT GGA AAT TGA TTA GTT CCT T-3′) and reverse primer COI_R (5′-AAA AAT TTT AAT TCC AGT TGG AAC AGC-3′) [34]. PCR was conducted according to the previously reported procedure of Chaiphongpachara et al. [35].

The PCR amplification of an approximately 300 bp fragment flanking codon 1014 of the *VGSC* gene was performed using two primers, including forward primer AgF_kdr (5′-GAC CAT GAT CTG CCA AGA TGG AAT-3′) and reverse primer An_kdr_R2 (5′-GAG GAT GAA CCG AAA TTG GAC-3′) [26]. The 25 µL PCR reaction consisted of 1U Platinum Taq DNA polymerase (Invitrogen, Carlsbad, CA), a 0.4 µM concentration of each primer, 1× reaction buffer, 1.5 mM MgCl_2_, 0.2 mM dNTPs, 4 μL of DNA template, and distilled water up to 25 μL. PCR amplifications were performed in a thermal cycler with the following temperature cycles: 94 °C at 5 min, 45 °C at 30 s, and 72 °C at 30 s, followed by 36 cycles of 94 °C at 30 s, 50 °C at 45 s, and 72 °C at 1 min; 35 cycles of 94 °C at 40 s, 54 °C at 60 s, and 72 °C at 1 min.

PCR amplification products of *COI* and *VGSC* were visualized on 1% agarose gels stained with Midori Green DNA stain (Nippon Gene, Tokyo, Japan) under an ImageQuant LAS 500 imager (GE Healthcare Japan Corp., Tokyo, Japan) for quality evaluation, before being sent to Solgent Company in Daejeon, South Korea, for the purification of PCR products and DNA sequencing.

### 2.6. Molecular Analyses

The trace files of *COI* and *VGSC* sequences for *Anopheles* specimens were manually aligned, checked, and edited, and consensus sequences were created from forward and reverse sequences using the BioEdit version 7.2 [36]. Afterward, *COI* and *VGSC* consensus sequences were aligned and manually edited using Clustal X [37] in the MEGA X (Molecular Evolutionary Genetics Analysis) software [38].

We compared the *COI* sequences of our *Anopheles* specimens to those available in GenBank to confirm species identification using the Basic Local Alignment Search Tool (BLAST, available online http://blast.ncbi.nlm.nih.gov/Blast.cgi (accessed on 10 October 2022) and the National Center for Biotechnology Information (NCBI) and BOLD (https://www.boldsystems.org/ (accessed on 10 October 2022) databases. Acceptance of *Anopheles* specimens required ≥98% nucleotide sequence identity for the available species sequences in the databases [39]. In addition, the intraspecific and interspecific genetic distances of all *Anopheles* species were calculated using the Kimura two-parameter distance algorithm (K2P) in MEGA X. We constructed a phylogenetic tree based on maximum likelihood (ML) with Tamura three-parameter plus gamma distribution plus invariable site model (best-fit substitution model) for *COI* sequences, using bootstrapping values defined for 1000 repetitions in MEGA X, in order to examine the evolutionary relationships among *Anopheles* species.

We used DNA Sequences Polymorphism (DnaSp) 6 software [40] to calculate the number of polymorphic (segregating) sites (s), nucleotide diversity (π), number of haplotypes (h), haplotype diversity (Hd), the average number of nucleotide differences (k), and statistical tests of neutrality, namely Tajima’s D test [41] and Fu’s *Fs* test [42], based on the mitochondrial *COI* gene, to investigate the genetic diversity of *Anopheles* mosquitoes in each species. In addition, haplotype networks of each *Anopheles* species were created using the median-joining network method in PopArt 1.7 to visualize the relationships among *Anopheles* individuals. For screening of *kdr* mutations in the *VGSC* gene, we investigated the *VGSC* sequences of all the specimens to find known resistant mutations (L1014C, L1014F, and L1014S).

## 3. Results

### 3.1. Anopheles Mosquitoes

In this study, 114 *Anopheles* females were caught in three geographical regions of Ranong province. Molecular identification revealed that the *Anopheles* specimens represented eight species, including *An. baimaii* (21.05%), *An. minimus* s.s. (20.17%), *An*. *epiroticus* (19.30%), *An*. *jamesii* (19.30%), *An. maculatus* s.s. (13.16%), *An*. *barbirostris* A3 (5.26%), *An. sawadwongporni* (0.88%), and *An. aconitus* (0.88%) (Table 1). Their distributions in Ranong province, as obtained in this study, are presented in Figure 1.

The southern part of Ranong had the highest number of *Anopheles* mosquito species (*n* = 7), followed by the northern (*n* = 4) and central (*n* = 2) parts, respectively. *Anopheles baimaii* was the only *Anopheles* species found across all three parts of Ranong province. In contrast, *An. aconitus* and *An. sawadwongporni* were extremely rare, with just a single specimen found in the southern and northern parts of the province, respectively. Thus, *An. aconitus* and *An. sawadwongporni*, represented by only one specimen each, were excluded from genetic diversity analyses.

### 3.2. Malaria Parasite Detection

According to the fast COX-I PCR method results, none of the 114 *Anopheles* mosquitoes examined were infected with *Plasmodium* parasites.

### 3.3. Nucleotide Sequences

One hundred and fourteen *COI* sequences of *Anopheles* mosquitoes were submitted to the GenBank database under accession numbers OP253978–OP254091 (Table 2) and were used for *COI* sequence analyses.

Intraspecific genetic divergences of *Anopheles* mosquitoes ranged from 0.2 to 1.3%, with an average value of 0.7% (Table 3). The highest intraspecific divergence was observed in *An. epiroticus* (1.3%), followed by *An. minimus* (1.1%), *An. baimaii* (0.5%), and *An. jamesii* (0.5%). In contrast, interspecific genetic divergences of *Anopheles mosquitoes* varied from 6.5 to 14.6%, with an average value of 10.7% (Table 3). The highest interspecific divergence was observed between *An. jamesii* and *An. barbirostris* A3 (13.6%), followed by those between *An. epiroticus* and *An. baimaii* (12%) and between *An. jamesii* and *An. epiroticus* (11.8%). Based on the genetic distances of all individuals, the maximum intra- and minimum interspecific genetic values were nonoverlapping; thus, all *Anopheles* species in this study could be correctly differentiated by *COI* sequence analysis.

### 3.4. Phylogenetic Analysis

**Figure 2:**
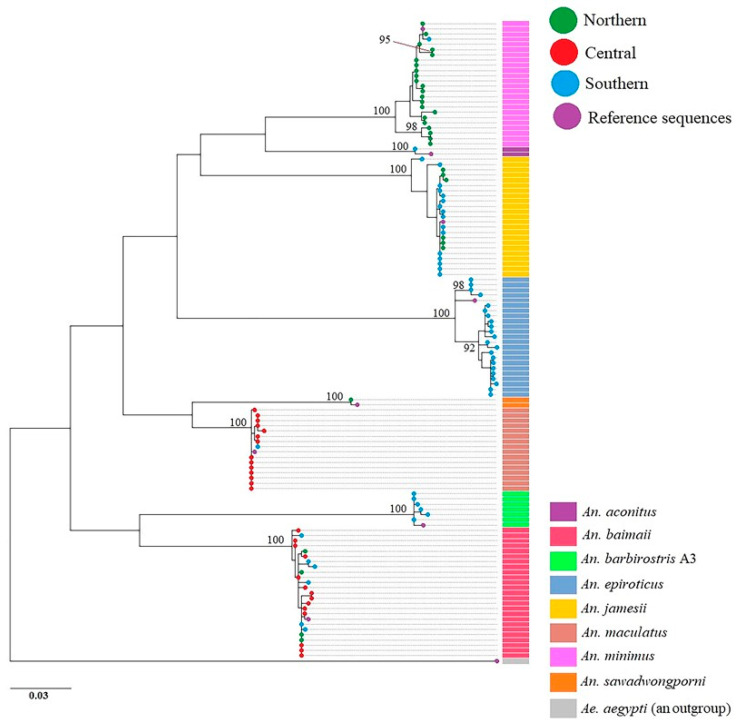
Maximum likelihood tree of eight *Anopheles* species identified in this study. Color bands indicate each species of *Anopheles* mosquito. The small colored circles represent the areas in which the samples were collected. Bootstrap values (1000 replicates) >90% are shown at the nodes. Eight sequences obtained from GenBank were used as species references, including *An. baimaii* (GenBank accession number: OL742839), *An. minimus* s.s. (OL742874), *An. epiroticus* (OL742858), *An. jamesii* (OL742865), *An. maculatus* s.s. (OL742869), *An. barbirostris* A3 (MT394436), *An. sawadwongporni* (OL742914), and *An. aconitus* (OL742831). Furthermore, *Aedes aegypti* (OL743100) was used as an outgroup in this analysis.

The ML phylogenetic tree showed that the species of *Anopheles* mosquitoes identified in this study was clearly separated into clades, supported by perfect bootstrap values (100%) (Figure 2). The *An. minimus* clade was sister to the *An. aconitus* clade, with the *An. jamesii* clade and the *An. epiroticus* clade far away, respectively. The *An. barbirostris* A3 and *An. baimaii* clades, which were sister clades, had the most distant relationships with other species. Sister clades of the *An. maculatus* group were positioned between sister clades of *An. barbirostris* A3 and *An. baimaii*, and the *An. epiroticus* clade. In addition, the phylogenetic analysis based on *COI* sequences indicated that the *An. minimus* and *An. epiroticus* clades were split into two distinct subclades and the *An. jamesii* clade was split into three distinct subclades.

### 3.5. Genetic Diversity

One hundred and twelve sequences were used to estimate the genetic diversity (the single sequences of *An. aconitus* and *An. sawadwongporni* were excluded). The nucleotide and haplotype diversity values of six *Anopheles* species are depicted in Table 4.

The genetic diversity index assessment showed that *An. epiroticus* had the highest level of nucleotide diversity (0.012 ± 0.001 SD), followed by *An. minimus* (0.011 ± 0.002 SD), *An. baimaii* (0.005 ± 0.001 SD)*, An. jamesii* (0.005 ± 0.001 SD)*, An. barbirostris* A3 (0.003 ± 0.001 SD), and *An. maculatus* (0.002 ± 0.000 SD) (Table 4). The highest level of haplotype diversity was observed in *An. epiroticus* (0.974 ± 0.022 SD/h = 17)*,* followed by *An. baimaii* (0.938 ± 0.039 SD/h = 17), *An. minimus* (0.925 ± 0.032 SD/h = 12), *An. jamesii* (0.900 ± 0.041 SD/h = 11), *An. barbirostris* A3 (0.800 ± 0.172 SD/h = 4), and *An. maculatus* (0.743 ± 0.090 SD/h = 5).

The Fu’s *Fs* and Tajima’s *D* values of six *Anopheles* species in Ranong were almost all negative, except Tajima’s *D* values of *An. minimus* (0.077, Table 4). These results suggested a high number of low-frequency mutations and that *Anopheles* populations in Ranong are undergoing demographic expansion. Significantly negative Fu’s *FS* values support this finding; however, Fu’s *FS* was significant in *An. baimaii, An. epiroticus,* and *An. jamesii*, whereas Tajima’s *D* was not significant in all species.

### 3.6. Haplotype Relationships

**Figure 3:**
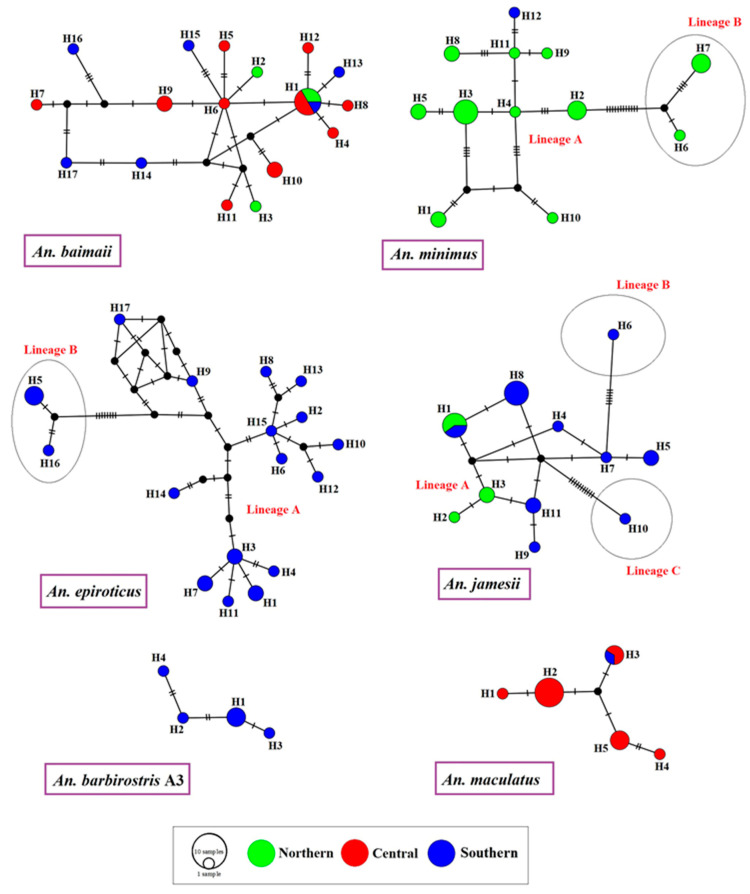
*COI* haplotype networks of *Anopheles* specimens collected from three locations in Ranong province, Southern Thailand. *Anopheles* species represented by only one specimen, including *An. aconitus* and *An. sawadwongporni*, were excluded from the analyses. A colored circle represents each haplotype, and the circle’s size is proportional to the total sequence of each haplotype. The number of mutations is shown by the dashes along the haplotype lines; the different colored circles represent different locations in Thailand.

The frequencies of and relationships between 112 haplotypes of *An. baimaii, An. barbirostris* A3, *An. epiroticus, An. jamesii, An. maculatus,* and *An. minimus* identified in Ranong based on *COI* sequences are shown in median-joining haplotype networks (Figure 3).

The network analysis of *An. baimaii* revealed that H1 was the central haplotype that was highly connected to haplotype lines, and was the only one found in all localities of Ranong (northern, central, and southern parts). The haplotype network of *An. minimus* showed two distinct genetic lineages, A and B, based on mutation steps on the haplotype lines, similar to the *An. epiroticus* network, which showed two lineages.

The central haplotype of *An. minimus* and *An. epiroticus* could not be identified because their frequencies were not clearly different. The haplotype network of *An. jamesii* showed that H8 was the most common haplotype and H1 was a shared haplotype between the northern and southern populations. Based on mutation steps, three genetic lineages of *An. jamesii* were identified. The haplotype networks of *An. barbirostris* A3 showed that H1 was the most frequent haplotype, and all haplotypes were connected in a straight line, whereas the *An. maculatus* network showed that H2 was the most frequent haplotype and H3 was the shared haplotype, including samples from the central and southern parts.

### 3.7. Screening VGSC-Mutation-Mediated Knockdown Resistance

**Figure 4:**
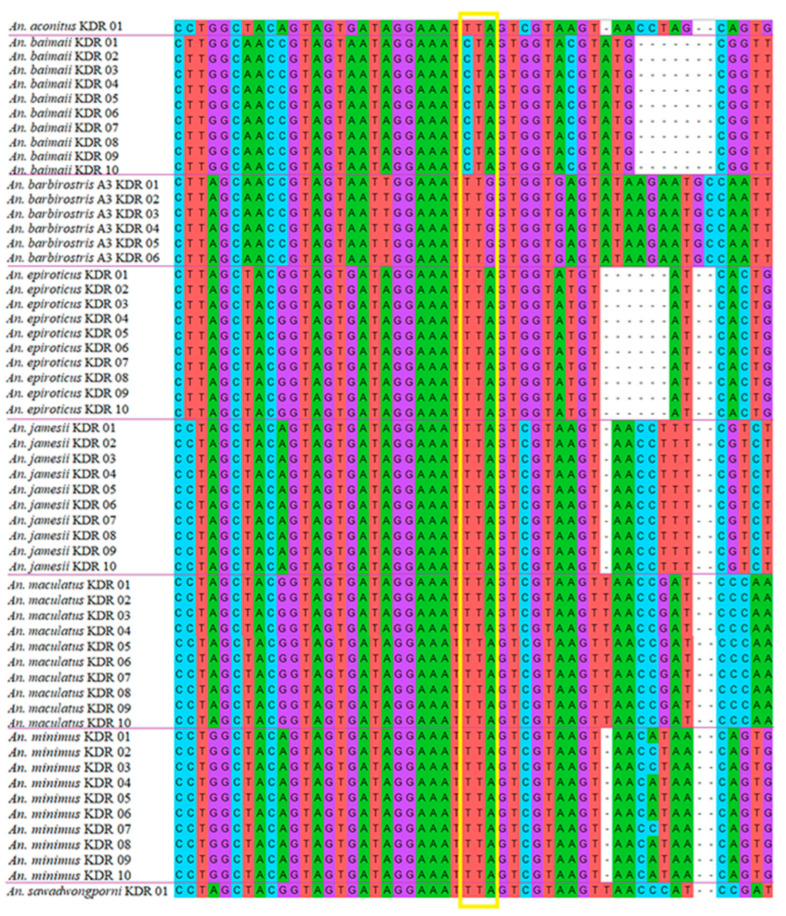
DNA sequences of a voltage-gated sodium channel gene fragment from specimens of the eight *Anopheles* species sampled in Ranong. The yellow rectangle shows the codon at the 1014 position, for which all samples presented an L1014 wild-type allele (TTA, TTG, or CTA).

One hundred and fourteen DNA sequences of the *VGSC* gene fragment from the *Anopheles* specimens were checked for screening of knockdown resistance mutations. All the sequenced specimens presented only the L1014 wild-type allele in the *VGSC* gene (Table 5). No *kdr*-resistant alleles (L1014C, L1014F, or L1014S) were found in any of the 114 *Anopheles* specimens screened (Figure 4, Table 5).

## 4. Discussion

In this study, specimens of eight species of *Anopheles* mosquitoes collected from Ranong province, which is Thailand’s most *P. knowlesi*-endemic area, were subjected to species confirmation using molecular methods; the species included *An. aconitus, An. baimaii, An. barbirostris* A3, *An. epiroticus, An. jamesii, An. maculatus* s.s., *An. minimus* s.s., and *An. sawadwongporni*. In the northern part of Ranong, a total of four *Anopheles* species were found: *An. baimaii, An. jamesii, An. sawadwongporni*, and *An. minimus* s.s. as the dominant species. In the central area, *An. baimaii* and *An. maculatus* s.s. were found to be the dominant species. Most *An. minimus* mosquitoes live in forest edge areas, so they are common in the northern part of Ranong where there are many forest edge areas. Meanwhile, the Hat Som Paen reservoir area, which consists of densely forested high mountains in the central part of Ranong, is a suitable habitat for *An. baimaii.* We also found *An. maculatus* in the reservoir area, which is likely their habitat, although previous reports indicated that they were predominantly distributed along the edge of the forest [15,16]. In the southern part of Ranong, *An. epiroticus* is the dominant species because their habitat is coastal areas.

Unfortunately, no *Anopheles* mosquitoes in this survey were infected with *Plasmodium* parasites. However, some species of *Anopheles* mosquitoes require special entomological surveillance, based on previous reports of *P. knowlesi* infections in other countries. Several previous studies in Malaysia have reported that *Anopheles* mosquitoes in the Leucosphyrus group are important vectors of *P. knowlesi* [43,44,45]. *Anopheles baimaii* (previously known as *An. dirus* species D) is a species member in the Dirus complex and belongs to the Leucosphyrus group [46]. This *Anopheles* species is considered the primary vector of human malaria in Thailand [47]. Our study results indicated that they are distributed in forested areas throughout Ranong. In addition, *An. nemophilous,* belonging to the Leucosphyrus group, has also been reported in Ranong province [48]. A previous study reported *P. knowlesi* infections in *An. sundaicus* s.l. in Katchal Island, India [14]. *Anopheles epiroticus* (previously known as *An. sundaicus* species A) is a common *Anopheles* species found near coastal areas in Ranong and other provinces of Thailand [49,50]. This *Anopheles* species belongs to the Sundaicus complex and is considered Thailand’s secondary vector of malaria [48,51]. *Anopheles barbirostris* species A3 is a cryptic species in the Barbirostris complex belonging to the Barbirostris subgroup [52]. In Thailand, this mosquito species has previously only been reported in Kanchanaburi province [52]. Our study is the first to demonstrate the additional distribution of *An. barbirostris* A3 in Thailand. However, *An. barbirostris* A3 is another species that should not be overlooked because *An. donaldi,* a member species in the Barbirostris subgroup, has been reported to carry *P. knowlesi* infections in Lawas, Northern Sarawak, Malaysian Borneo [9]. In addition, investigations of blood meal sources of malaria vector mosquitoes using specific PCR assays should be continued in the future to determine their anopheline anthropophilic, zoophilic, or zoo-anthropophilic origin. The host preferences of *Anopheles* species are very important pieces of information in evaluating their ability to transmit simian malaria to humans [53].

The genetic distances between the eight *Anopheles* taxa based on 114 *COI* sequences showed that the maximum intra- and minimum interspecific genetic values were nonoverlapping, indicating the existence of a distinct barcode gap. The presence of a barcoding gap confirms the success of DNA barcoding for species identification [54]. Recently, Chaiphongpachara et al. [35] succeeded in identifying several mosquito species in Thailand based on nucleotide differences in the *COI* gene, except for *An. dirus* and *An. baimaii*. Our study results provide supporting evidence that DNA barcoding based on *COI* can be used to identify mosquito species. However, other DNA markers, such as ITS2, must be used for species identification in cases where *Anopheles* mosquitoes in the Dirus complex are found [55].

The nucleotide diversity (π) and nucleotide diversity (Hd) are important genetic indicators used to measure genetic diversity among populations. The nucleotide diversity values of *An. baimaii, An. barbirostris* A3, *An. epiroticus, An. jamesii, An. maculatus*, and *An. minimus* in Ranong were lower than the haplotype diversity values, indicating a recent *Anopheles* population expansion to a small effective population size after a bottleneck [56]. This demographic event occurred long enough ago for the haplotypes to increase through mutation, but not enough for the accumulation of large sequence differences [57]. Furthermore, a high level of haplotype diversity results from a large population and different environments and living habits suitable for their rapid development in nature [58].

Our results showed that genetic diversity values were similar in some species and different in others, for multifactorial reasons. Mosquito genetic diversity has both internal and external causes [59]. Internal causes of genetic diversity are genetic mutations or changes, whereas external factors are strongly related to the ecological environment of the mosquitoes [59]. Genetic diversity is an important factor that allows natural populations to adapt to and survive long-term changes or adverse environmental conditions [60]. Ranong is one of the provinces in southern Thailand with unique ecological features. The area is covered by mountains and fertile forests, and is adjacent to the Andaman Sea. It is also the wettest province in Thailand. It has been previously reported that *Anopheles* mosquito populations can swiftly adapt to alterations in environmental conditions, which may impact the genetic diversity within species at the population level and their gene flow [56,61]. However, a limitation of our study was the assessment of *Anopheles* vectors in only one endemic area, which provided insufficient data for this answer.

The Fu’s *Fs* and Tajima’s *D* values of *An. baimaii, An. barbirostris* A3, *An. epiroticus, An. jamesii*, and *An. maculatus* in Ranong all showed negative values, supporting population size expansion in Ranong. If a population is selectively neutral and at equilibrium between genetic drift and selectively neutral mutation, the Tajima’s *D* value is expected to be zero. Positive Tajima’s *D* values indicate a sudden decrease in population size and/or balancing selection, whereas negative Tajima’s *D* values indicate population size expansion after a recent bottleneck or mutational selection [41]. Our results were consistent with the population structure of *An. baimaii* in Thailand, indicating that the population is expanding [56].

The ML phylogenetic tree and haplotype network results revealed two distinct genetic lineages, A and B, of *An. minimus* and *An. epiroticus* in Ranong. Recently, Bunmee et al. [56] reported the existence of A and B lineages for *An. minimus* s.s. in Thailand, which agrees with our research findings. In many of Thailand’s malaria transmission areas, such as Tak, Surat Thani, Yala, Chanthaburi, and Trat, there are two lineages of *An. minimus*, which are often found together [56]. However, it is unclear whether the two lineages have the potential to transmit malaria or other different behaviors. In addition, our study is the first to reveal two distinct genetic lineages of *An. epiroticus* based on the *COI* gene, which shows genetic variation and local adaptation. Syafruddinid et al. [62] explained that the *COI* gene is suitable for assessing genetic variation within populations of *An. epiroticus* because mtDNA has a high mutation rate. However, this gene cannot be used as a molecular marker to differentiate between *An. epiroticus* and its other sibling members [62]. Although three genetic lineages of *An. jamesii* were identified, only one group had many samples, whereas the other two groups had only one member each. Consequently, future genetic studies on this species should be conducted.

The early detection and surveillance of *VGSC*-mutation-mediated knockdown resistance (*kdr*) in *Anopheles* populations can provide entomological data on the causes of pyrethroid resistance in insects to inform the development of strategies to control malaria vectors [63]. The present study showed no presence of *kdr* mutation in the *VGSC* gene among *Anopheles* mosquitoes from Thailand’s most *P. knowlesi*-endemic area. This result is similar to those of previous *Anopheles* investigations in Ubon Ratchathani province, northeastern Thailand [64]. However, this study is limited by a lack of information on the susceptibility of the mosquito samples tested. Therefore, we do not know the true state of insecticide resistance in these *Anopheles* populations. Further entomological investigations into the susceptibility of adult mosquito vectors to insecticides are required.

## 5. Conclusions

Our study demonstrated the genetic diversity of *Anopheles* mosquitoes in Thailand’s most *P. knowlesi*-endemic area. Our genetic diversity analysis will contribute to a more comprehensive genetic profile of *Anopheles* vectors in Thailand. In addition, our attempts to detect *P. knowlesi* infection in *Anopheles* mosquitoes did not reveal infected specimens. However, three *Anopheles* species, including *An. baimaii, An. barbirostris* A3, and *An. epiroticus,* should be kept under special surveillance as *P. knowlesi* infections have been found in these species in other countries. This entomological information could lead to the selection of appropriate methods for controlling these *Anopheles* populations, such as insecticide-treated nets (ITNs) and indoor residual spraying (IRS), in order to control the spread of monkey malaria to humans in Thailand’s most *P. knowlesi*-endemic area. In addition, educating the population about vector breeding sites and strategies for protection against *Anopheles* mosquitoes, such as applying mosquito repellent or wearing protective clothes when entering forests where monkeys reside, are crucial ways to help reduce the incidence of knowlesi malaria.

## Figures and Tables

**Table 1:** Species and numbers of *Anopheles* sampled in the geographical region of Ranong province, Thailand (January–June 2022), identified using molecular methods.

*Anopheles* Species	Ranong Province			Total (%)
	Northern Part	Central Part	Southern Part	
*An. aconitus*	0	0	1	1 (0.88)
*An. baimaii*	4	14	6	24 (21.05)
*An. barbirostris* A3	0	0	6	6 (5.26)
*An. epiroticus*	0	0	22	22 (19.30)
*An. jamesii*	6	0	16	22 (19.30)
*An. maculatus* s.s.	0	14	1	15 (13.16)
*An. minimus* s.s.	22	0	1	23 (20.17)
*An. sawadwongporni*	1	0	0	1 (0.88)
Total (%)	33 (28.95)	28 (24.56)	53 (46.49)	114 (100)

**Table 2:** GenBank accession numbers of *COI* sequences obtained in this study.

*Anopheles* Species	*n*	Location	GenBank Accession Numbers
*An. Aconitus*	1	Southern part of Ranong	OP253978
*An. Baimaii*	4	Northern part of Ranong	OP253979–OP253982
	14	Central part of Ranong	OP253983–OP253996
	6	Southern part of Ranong	OP253997–OP254002
*An. barbirostris* A3	6	Southern part of Ranong	OP254003–OP254008
*An. epiroticus*	22	Southern part of Ranong	OP254009–OP254030
*An. jamesii*	6	Northern part of Ranong	OP254031–OP254036
	16	Southern part of Ranong	OP254037–OP254052
*An. maculatus*	14	Central part of Ranong	OP254053–OP254066
	1	Southern part of Ranong	OP254067
*An. minimus*	22	Northern part of Ranong	OP254068–OP254089
	1	Southern part of Ranong	OP254090
*An. sawadwongporni*	1	Northern part of Ranong	OP254091

**Table 3:** Interspecific and intraspecific K2P genetic distances of *Anopheles* species were collected based on *COI* sequences in this study.

*Anopheles* Species	% Mean Sequence Divergence (Min–Max)							
	1	2	3	4	5	6	7	8
1. *An. aconitus*	NA							
2. *An. baimaii*	11.5% (11.1–11.7)	0.5% (0.0–1.3)						
3. *An. barbirostris* A3	11.1% (10.9–11.2)	9.6% (9.3–10.1)	0.3% (0.0–0.7)					
4. *An. epiroticus*	10.7% (9.9–11.2)	12% (10.9–12.9)	11.6% (10.9–12.4)	1.3% (0.0–2.7)				
5. *An. jamesii*	10% (9.8–10.4)	11.3% (10.7–12.4)	13.6% (13.1–14.6)	11.8% (11.1–12.6)	0.5% (0.0–1.9)			
6. *An. maculatus*	10.2% (10.1–10.4)	8.9% (8.3–9.8)	10.2% (9.9–10.4)	11.6% (10.8–12.4)	9.5% (9.3–9.8)	0.2% (0.0–0.7)		
7. *An. minimus*	8.2% (7.9–8.4)	10.9% (10.4–11.2)	11.2% (10.6–11.6)	11.5% (10.6–12.7)	11% (10.6–12.1)	10% (9.5–10.4)	1.1% (0.0–2.9)	
8. *An. sawadwongporni*	10.6% (10.6–10.6)	11.5% (11.2–11.9)	10.6% (10.4–10.7)	11.5% (10.9–11.9)	11.7% (11.6–12.1)	6.6% (6.5–6.6)	9.9% (9.5–10.3)	NA

**Table 4:** Genetic diversity indices and neutrality tests of *Anopheles* species in Ranong.

*Anopheles* Species	*n*	s	π (±SD)	h	Hd (±SD)	k	Neutrality Tests	
							Fu’s *Fs*	Tajima’s *D*
*An. aconitus*	1	–	–	1	–	–	–	–
*An. baimaii*	24	22	0.005 ± 0.001	17	0.938 ± 0.039	3.616	−10.476 *	−1.621
*An. barbirostris* A3	6	5	0.003 ± 0.001	4	0.800 ± 0.172	2.067	−0.439	−0.315
*An. epiroticus*	22	33	0.012 ± 0.001	17	0.974 ± 0.022	8.797	−4.804 *	−0.220
*An. jamesii*	22	20	0.005 ± 0.001	11	0.900 ± 0.041	3.667	−2.544 *	−1.233
*An. maculatus*	15	6	0.002 ± 0.000	5	0.743 ± 0.090	1.695	−0.214	−0.285
*An. minimus*	23	29	0.011 ± 0.002	12	0.925 ± 0.032	8.016	−0.211	0.077
*An. sawadwongporni*	1	–	–	1	–	–	–	–

*Anopheles aconitus* and *An. sawadwongporni,* represented by only one specimen each, were excluded from the analyses. An asterisk (*) after Fu’s *Fs* and Tajima’s *D* values represents the statistical difference at *p* < 0.05. Abbreviations: *n* = number of sequences; s = number of polymorphic (segregating) sites; π = nucleotide diversity; h = number of haplotypes; Hd = haplotype diversity; k = average number of nucleotide differences.

**Table 5:** Screening for *kdr* mutations in the voltage-gated sodium channel (*VGSC*) gene in specimens of eight *Anopheles* species obtained in this study.

Species	Allelic Frequency					
	L1014 Wild			L1014C (TGT)	L1014F (TTT)	L1014S (TCA)
	TTA	TTG	CTA			
*An. Aconitus*	1	0	0	0	0	0
*An. baimaii*	0	0	24	0	0	0
*An. barbirostris* A3	0	6	0	0	0	0
*An. epiroticus*	22	0	0	0	0	0
*An. jamesii*	22	0	0	0	0	0
*An. maculatus*	15	0	0	0	0	0
*An. minimus*	23	0	0	0	0	0
*An. sawadwongporni*	1	0	0	0	0	0
Total	84	6	24	0	0	0

## Data Availability

Not applicable.
